# Patient Decision Aids for Pelvic Floor Surgery and Impact on Decisional Conflict

**DOI:** 10.1111/1471-0528.18311

**Published:** 2025-07-28

**Authors:** Roberta Bugeja, Ruth Athey, Swati Jha

**Affiliations:** ^1^ Sheffield Teaching Hospitals NHS Foundation Trust Sheffield South Yorkshire UK; ^2^ University of Sheffield Sheffield South Yorkshire UK

**Keywords:** decisional conflict scale, decision‐making, patient decision aid, PDA, stress urinary incontinence, uterine prolapse, vault prolapse

## Abstract

**Objectives:**

To evaluate whether the use of a patient decision aid (PDA) diminishes decision conflict in patients considering surgical treatment for stress urinary incontinence (SUI) or pelvic organ prolapse (POP) and to assess patient satisfaction concerning the PDA's user‐friendliness and overall utility.

**Design:**

One hundred women attending routine urogynaecology clinic appointments were offered the PDA as part of standard care and recruited into the study. After using the PDA, participants completed a decisional conflict scale (DCS) to assess their decision‐making experience.

**Setting:**

Single‐site NHS tertiary‐level care hospital outpatient department.

**Population and Sample:**

The study included women considering surgical management for uterine/vault prolapse or SUI. Exclusion criteria comprised individuals under 18 years of age, those unable to communicate in English and patients not eligible for all surgical options presented in the PDAs.

**Methods:**

Patients presenting to the urogynaecology clinic with symptomatic SUI or POP considering surgical treatment were invited to participate in the study. After using the PDA and at their next clinic visit, participants completed a DCS to assess their decision‐making experience.

**Main Outcome Measure:**

The DCS assessed the level of uncertainty and difficulty participants experienced in deciding on surgical treatment after using the PDA. A DCS score of less than 25 was associated with certainty in implementing the decision.

**Results:**

This was a prospective cohort study of 100 participants. The DCS scores for the cohorts showed low decisional conflict, with median scores of 0.8 (IQR 4.7) for the vault PDA, 6.3 (IQR 25) for the uterine PDA and 7.8 (IQR 21.1) for the SUI PDA. A sub‐score analysis revealed higher scores for ‘uncertainty’ and ‘effective decision making’ in 21 participants with total DCS scores above 25.

**Conclusion:**

NICE PDAs are valuable tools for enhancing decision‐making in gynaecological surgery. However, this study highlights the need for their ongoing refinement to better address the informational and emotional aspects of patient decision‐making. Future research should focus on incorporating emotional support frameworks and psychological management tools to improve their clinical utility and effectiveness.

## 1 Introduction

A key challenge in urogynaecology is ensuring active patient involvement in decision‐making, especially when selecting appropriate treatment options [1].

In 2019, the National Institute for Health and Care Excellence (NICE) published guidance on the management of stress urinary incontinence (SUI) and pelvic organ prolapse (POP), which included the introduction of patient decision aids (PDAs) to assist in discussions regarding surgical options for SUI, uterine prolapse and vaginal vault prolapse [2].

PDAs have emerged as valuable tools. They aim to support shared decision‐making between healthcare providers and patients. These aids also educate patients about their condition, outline available treatment options and help them integrate personal preferences and values into their decision‐making process [3].

The application of PDAs in urogynaecology can offer multiple benefits. These aids can be provided in various formats, including self‐directed tools for use outside clinical settings or interactive resources utilised during consultations with healthcare providers. By delivering comprehensive information on the benefits and risks of treatment options, PDAs enable patients to make informed choices that reflect their individual needs and preferences.

Despite the inclusion of PDAs in NICE guidelines, evidence of their effectiveness in the context of pelvic floor dysfunction remains scarce. Studies on alternative PDAs for SUI suggest promising outcomes. For example, a PDA developed by Jha et al. (2019) demonstrated high effectiveness, with 95% of participants reporting confidence in their decision [4]. Similarly, another study found that patients using a PDA developed by their research team exhibited low decision conflict, with a mean decisional conflict scale (DCS) score of 9.29, indicating a high confidence level in their decision [5]. When the NICE PDAs were launched into clinical practice, they had not been tested in clinical practice, also referred to as Beta testing of a PDA. So, at the point of introduction, they were not validated.

Therefore, the effectiveness of NICE PDAs in decision‐making for SUI, uterine or vaginal vault prolapse surgery has yet to be thoroughly investigated, and additional evidence is needed to assess their impact in this context.

## 2 Methods

The aim of this study was to determine whether the use of the PDAs, recommended by NICE for the surgical management of SUI, uterine prolapse and vaginal vault prolapse, improved shared decision‐making and patient satisfaction. The DCS was used to assess patients' perceptions of the quality of information, clinical and personal uncertainty, alignment with their values, support from the clinical team and overall satisfaction with the decision‐making process.

This prospective observational study was conducted in the urogynaecology clinic at a tertiary NHS hospital. Ethics approval for a two‐part study was obtained. A qualitative study in which semistructured interviews of women using each of the 3 PDAs was undertaken with a clinical researcher, and the results of these are presented elsewhere. The study presented here is the quantitative element of the study. Eligible participants were women with symptomatic SUI, uterine prolapse or vaginal vault prolapse who were considering surgical management. Participants were invited by their clinicians, provided with written information about the study, and consented to participation prior to data collection. Demographic data, including literacy levels and relevant medical information, were collected from patient records and clinical questioning. All patients were invited back to the clinic once they had an opportunity to review the PDA to discuss their surgical procedure of choice. This was part of routine clinical practice. It was at this consultation and following the use of the PDA that participants completed the DCS.

No control group was included because NICE guidelines mandate using PDAs in all cases. The primary outcome was whether using the PDA led to low levels of decisional conflict and improved confidence for patients in the decision‐making process.

The sample size calculation was carried out based on the authors’ previous experience of validation of a PDA for pelvic floor surgery. Assuming a one‐point mean difference is of clinical and practical importance, to have a 95% power of detecting a 1‐point mean difference in the DCS at the 5% (two‐sided) level would require a minimum of 30 patients in each group. A sample size of 105 patients was agreed to allow for a dropout rate.

One hundred and five women were recruited in the study, of which 100 completed the DCS. Five women withdrew from the study. Thirty participants received the PDA for surgical management of vault prolapse, and 35 participants received the PDA for surgical management of SUI and uterine prolapse respectively. For each cohort, the range of DCS scores was calculated to provide an understanding of score dispersion. The interquartile range (IQR), defined as the difference between the first (Q1) and third (Q3) quartiles, was then computed to quantify the dispersion of the central 50% of scores, providing a more robust measure of variability less influenced by outliers.

Each unit increase in the DCS is associated with substantially higher odds of negative decision‐related outcomes, such as a future change in decision, delay in decision making, decisional regret and clinician blame in the event of a poor outcome.

The DCS (Figure S1) used to assess decision conflict is a 16‐question survey divided into five subsections, enabling the calculation of an overall decision conflict score and the identification of specific areas of increased conflict [6]. The five subsections are as follows:

1. Informed (Questions 1, 2, 3): This subscale measures the patient's perception of how well they understand the information provided about their treatment options, including the potential risks and benefits.
2. Values Clarity (Questions 4, 5, 6): This subscale assesses the degree to which patients feel that their personal values and preferences have been clearly identified and incorporated into the decision‐making process.
3. Support (Questions 7, 8, 9): This subscale examines the perceived support the patient receives from healthcare providers, family and friends in making the decision, addressing emotional and informational support.
4. Uncertainty (Questions 10, 11, 12): This subscale evaluates the level of uncertainty the patient experiences regarding the decision at hand. It includes items related to confusion about available options and the perceived difficulty of deciding.
5. Effective Decision (Questions 13, 14, 15, 16): This subscale evaluates the patient's confidence and satisfaction with the decision, focusing on whether it seems effective and aligns with their values.

Each item within the scale is rated on a 5‐point Likert‐type scale, with responses ranging from ‘strongly agree’ to ‘strongly disagree’ which are numerically scored from 0 to 4. The scores from the individual items within each subscale are averaged to generate the subscale score. These subscale scores and the total DCS score provide a detailed overview of the patient's decision conflict across various dimensions [6].

To calculate the total score, the mean score for each question was determined by summing the scores and dividing by 16, then multiplying by 25 to obtain a value between 0 and 100. This method, previously validated and used by the Cochrane Database, indicated that a score of 25 or less was associated with low decision conflict and high satisfaction. In contrast, a score of 37.5 or higher indicated delays or uncertainty in decision‐making [6].

If the total conflict score for a participant was high, scores for each subsection were also calculated to identify specific areas contributing to the decision conflict. The mean score for each subsection was calculated similarly to the total score, then multiplied by 25 to yield a value between 0 and 100. This provided initial insight into areas of the PDA that may require improvement.

**FIGURE 1:**
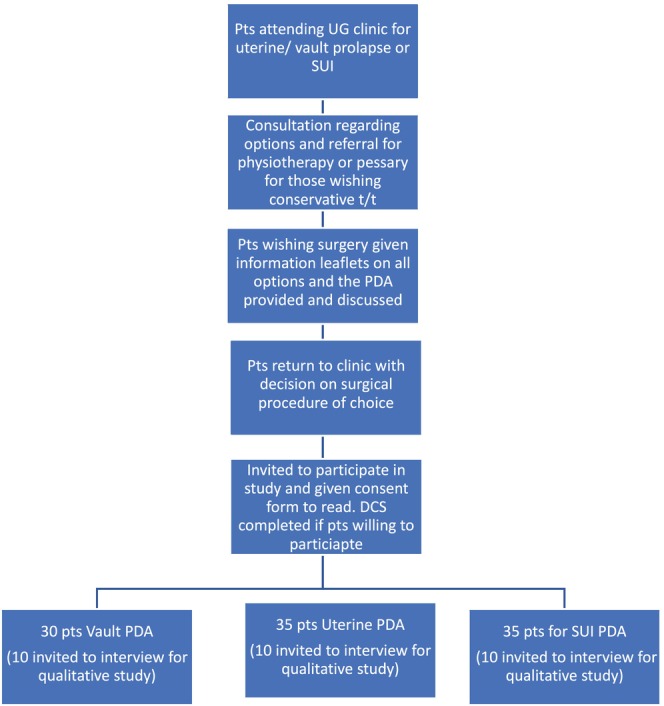
Patient flow diagram.

The flow of patients through the study is shown in Figure 1.

## 3 Results

The demographics of participants for each cohort were reported (Table 1). The mean age for the cohort receiving the vault PDA was 63 years (range 43–76); 59 years for the uterine PDA (range 36–80 years) and 48 years for the SUI PDA (range 32–76 years). All participants reported English as their preferred language for communication. Some participants were still undecided about their preferred surgical management for POP or SUI at the time of completion of the DCS: five patients in the uterine prolapse and in the vault prolapse cohort and 10 patients in the SUI group.

**TABLE 1:** Patient Demographics.

	Vault (*n* = 30)	Uterine (*n* = 35)	SUI (*n* = 35)
Mean age	63 (Range 43–76)	59 (Range 36–80)	48 (Range 32–76)
Preferred language	English (100%)	English (100%)	English (100%)
Choice of procedure	Sacrospinous fixation (40%)	Vaginal hysterectomy (86%)	Autologous fascial sling (34%)
	Sacrocolpopexy (40%)	Undecided (14%)	Colposuspension (8%)
	Colpocleisis (3%)		Periurethral bulking agent injection (29%)
	Undecided (17%)		Undecided (29%)

The DCS scores of participants in the three cohorts were reported (Table 2). The median score reported for participants using the vault PDA was 0.8 (IQR 4.7), 6.3 (IQR 25) for those using the uterine prolapse PDA and 7.8 (IQR 21.1) for those using the PDA for SUI.

**TABLE 2:** Decisional Conflict Scale (DCS) Scores.

	Vault prolapse PDA (*n* = 30)	Uterine prolapse PDA (*n* = 35)	SUI PDA (*n* = 35)
Median score	0.8	6.3	7.8
Range	28.1	46.9	100
Interquartile range (IQR)	4.7	25	21.1

A sub‐score analysis was performed for the participants whose total DCS score was 25 or above (Table 3). One participant was in the vault prolapse cohort, 11 in the uterine prolapse group and nine in the SUI group. Seven (7/21) of these participants indicated that they were still unsure about the surgical management option for treating their pelvic floor condition when completing the DCS. Higher sub‐scores were seen in the questions corresponding to ‘uncertainty’ and ‘effective decision making’.

**TABLE 3:** Decisional Conflict Scale (DCS) sub‐scores.

		Informed [1,2,3]	Values clarity [4,5,6]	Support [7,8,9]	Uncertainty [10,11,12]	Effective decision [13,14,15,16]
Vault prolapse PDA (*N* = 1)	Median score	4.7	4.7	6.3	6.3	6.3
	Interquartile range (IQR)	—	—	—	—	—
Uterine prolapse PDA (*N* = 11)	Median score	4.7	4.7	4.7	6.3	6.3
	Interquartile range (IQR)	0.8	0.0	0.0	4.7	6.3
SUI PDA (*N* = 9)	Median score	4.7	6.3	4.7	9.4	7.8
	Interquartile range (IQR)	3.1	4.7	1.6	5.5	6.3

*Note:* The numbers within brackets refers to the question number in the DCS (Figure S1) which is used to calculate the respective scores.

## 4 Discussion

### 4.1 Main Findings

The study demonstrates that the median DCS scores for all three PDAs were below 25, with narrow interquartile ranges. As a DCS score of 25 or lower indicates low decisional conflict, this confirms that participants experienced a high degree of satisfaction and certainty when making their surgical treatment decisions.

This finding is consistent with prior research that found a significant reduction in decision conflict when PDAs are used in clinical settings, particularly in complex medical decisions such as pelvic floor surgery [3, 4]. The observed low DCS scores across all cohorts in this study provide strong evidence of the effectiveness of PDAs in facilitating shared decision‐making and improving patient confidence in the choices made. By lowering decisional conflict, PDAs may also help mitigate feelings of regret or dissatisfaction arising from surgical decisions, as patients feel more informed and empowered in their choices. This is consistent with findings from other studies, which reported that patients who used PDAs for surgical decision‐making were more likely to feel their decisions aligned with their values and preferences and thus were less likely to experience post‐decision regret [5].

## 5 Strengths and Limitations

The development of a PDA is a process that involves alpha and beta testing followed by quantitative analysis using the Ottawa Decision Conflict scale [7, 8, 9]. The NICE PDA was not developed according to these stringent methodologies due to the constraints of time and urgent publication following recommendations by the Cumberlege Review and Report [10]. However, they have been implemented into practice without any evidence of their efficacy, and a failure to use them could be construed by litigation agencies as substandard clinical care.

This is the only study to evaluate the utility of these NICE PDAs which now constitute routine clinical practice in the UK. Robust methodologies have been used for testing and the tools used for assessment of the PDA are in accordance with processes developed and approved by Cochrane [6]. The sample size was appropriate and the age distribution among participants in the three cohorts was sufficient to capture a good age range.

The limitation of the study is that the PDA are only in the English language and therefore it was not possible to test them in a more diverse population of women for whom English was not their first language. The barriers to consent even with an interpreting service have been widely established [11], so the patients who may most benefit from the use of the PDA could not be included.

Another limitation of the study is that it is difficult to eliminate the role of the clinician in the decision‐making process and assumes that all clinicians communicate in the same standardised manner. It also fails to exclude the role of other interventions such as written patient information, verbal discussions and digital interventions also used in the consent process which have been shown to be central to the decision‐making process [12].

Despite having read the PDA and having had a consultation, 20/100 participants were still undecided about the surgical option for treatment of their condition. It may have been helpful to undertake further interviews with these patients specifically to understand why they remained undecided when the interventions in all patients were similar.

## 6 Interpretation (In Light of Other Evidence)

Surgical decision‐making for gynaecological patients, particularly those considering interventions for stress urinary incontinence (SUI) and pelvic organ prolapse (POP), is inherently complex and multifactorial, even with the use of patient decision aids (PDAs). In this study, further analysis of the DCS sub‐scores was conducted for participants who scored 25 or above, indicating higher levels of decisional conflict. Interestingly, of the 21 participants who had a total DCS score of 25 or higher, 14 had already consented to a surgical procedure for either POP or SUI. This suggests that, despite the use of the PDA, some patients continued to experience significant conflict or uncertainty regarding their decision, even after committing to surgery. The scores were higher in the questions contributing to uncertainty in their decision and confidence and satisfaction with the decision made. This finding aligns with previous research, which found that some patients, despite using PDAs, still reported residual uncertainty, particularly around the long‐term risks of surgery or alternative treatment options [5]. Additionally, although PDAs effectively improve patient knowledge and reduce some forms of decision conflict, they may not fully address emotional concerns or factors, such as the perceived pressure from clinicians or family members, which can influence decision‐making [3]. Therefore, while PDAs may aid in providing information, some patients may still experience decisional conflict, particularly those who have already decided but continue to struggle with underlying uncertainties.

However, the study also highlights important nuances in the decision‐making process. Despite the low overall DCS scores, a notable proportion of participants remained undecided about their treatment options, and some participants who scored high on the DCS had already consented to surgery. These findings suggest that, while PDAs are valuable tools in providing information, they may not fully address the emotional and psychological aspects of decision‐making, particularly for patients facing high‐stakes decisions like surgery. Furthermore, the higher DCS scores in subscales related to uncertainty and satisfaction with the decision made suggest that some patients may require additional support in navigating these emotional and cognitive dimensions of decision‐making.

## 7 Conclusion

In conclusion, this study provides compelling evidence that the use of NICE‐recommended PDAs effectively is associated with low decisional conflict among patients considering surgical management for POP and SUI. The overall low DCS scores across all cohorts suggest that the PDAs facilitated shared decision‐making, enhancing patient satisfaction and confidence in their surgical choices. These findings align with previous studies, demonstrating that PDAs significantly improve decision quality and reduce uncertainty in complex medical decisions [3, 4]. Additionally, the low decisional conflict in this study provides further validation of the role of PDAs in empowering patients, allowing them to make informed choices that align with their values and preferences, thereby mitigating potential post‐decision regret.

While PDAs are a valuable tool in improving decision‐making in gynaecological surgery, given there were patients in each group who remained undecided about their choice of surgery, the findings of this study underscore the need for ongoing refinement of these aids to better support patients in both the informational and emotional aspects of their decision‐making journey. This was further substantiated by the qualitative element of this study which has been previously published [13]. Future research should focus on integrating additional components into PDAs such as emotional support frameworks or tools to manage the psychological aspects of surgical decisions better to enhance their utility and effectiveness in clinical practice.

## Author Contributions

R.B.: Acquisition, analysis and interpretation of data. Manuscript authorship. R.A.: Acquisition of data. S.J.: Conception and development of project. Acquisition, analysis and interpretation of data. Manuscript authorship, review and approval.

## Ethics Statement

IRAS ID 313282, REC 22/PR/0414. IRAS/HRA approval and local ethical approval.

## Conflicts of Interest

The authors declare no conflicts of interest.

## Supporting information


Supplementary Figure S1.

## Data Availability

Data sharing not applicable to this article as no datasets were generated or analysed during the current study.
