# Supplementary material for: Patient Decision Aids for Pelvic Floor Surgery and Impact on Decisional Conflict
Source: BJOG. 2025 Jul 28;132(12):1817–22. doi: 10.1111/1471-0528.18311 (PMC12501710; doi:10.1111/1471-0528.18311)

Appendix 1: Decision Conflict Scale (DCS) (6)

A: Which option do you prefer:

(The Options available in each PDA were listed together with unsure)

B. Considering the option you prefer, please answer the following Questions:


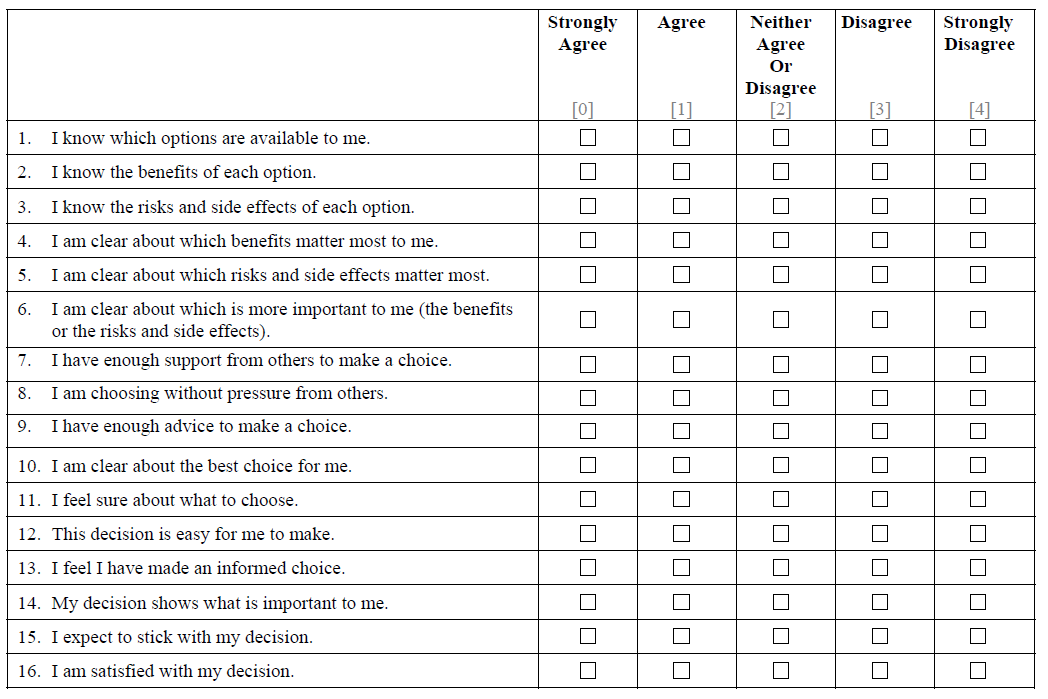

Supplement: Supplementary file 1 — Supplementary Figure S1. [file BJO-132-1817-s001.docx]
